# Gastrointestinal Anisakidosis – Watch What You Eat

**DOI:** 10.7759/cureus.860

**Published:** 2016-11-02

**Authors:** Moiz Ahmed, Faez Ayoob, Mayurathan Kesavan, Vivek Gumaste, Ambreen Khalil

**Affiliations:** 1 Department of Internal Medicine, Staten Island University Hospital; 2 Department of General Surgery, Staten Island University Hospital; 3 Department of Gastroenterology, Staten Island University Hospital; 4 Department of Infectious Disease, Staten Island University Hospital

**Keywords:** anisakiasis, peritonitis

## Abstract

Gastrointestinal anisakidosis is an under-reported and often misdiagnosed parasitic infection caused by the larvae of a nematode anisakis. The majority of cases are seen in Japan due to the consumption of raw and undercooked seafood; however, the incidence is likely to rise in the United States given the rising popularity of Japanese cuisine like sashimi or sushi. This unique report highlights the importance of considering anisakiasis in the differential diagnoses for patients with nonspecific abdominal symptoms with a recent history of raw or undercooked fish consumption.

## Introduction

Human anisakiasis begins with the accidental ingestion of Anisakis simplex larvae by consuming raw or undercooked fish or marine mollusks such as squids, octopus and cuttlefish. These larvae mostly invade and penetrate the stomach, and can be easily seen on upper endoscopy [1]. However, enteric and ectopic anisakiasis is rare and often a challenge to diagnose due to its nonspecific nature of abdominal symptoms. Diagnosis usually requires invasive surgical exploration due to intestinal obstruction, peritonitis or intestinal perforation. In a study involving 201 cases of bowel anisakiasis in Japan, where 56% had abdominal complications, only seven percent required open surgery, indicating a significant role for conservative therapy [2]. Here we report a rare case of acute abdominal peritonitis due to penetration of the gastrointestinal tract by the Anisakis simplex larvae.

Informed consent was obtained from the patient for the procedure.

## Case presentation

**Figure 1:**
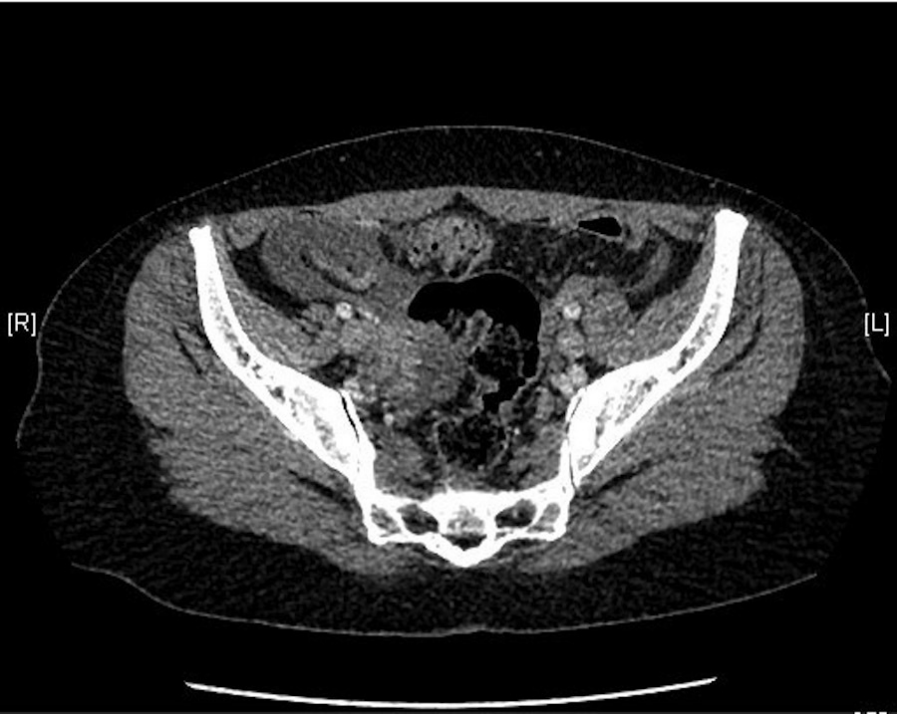
Abdominal CT image showing free pelvic fluid

**Figure 2:**
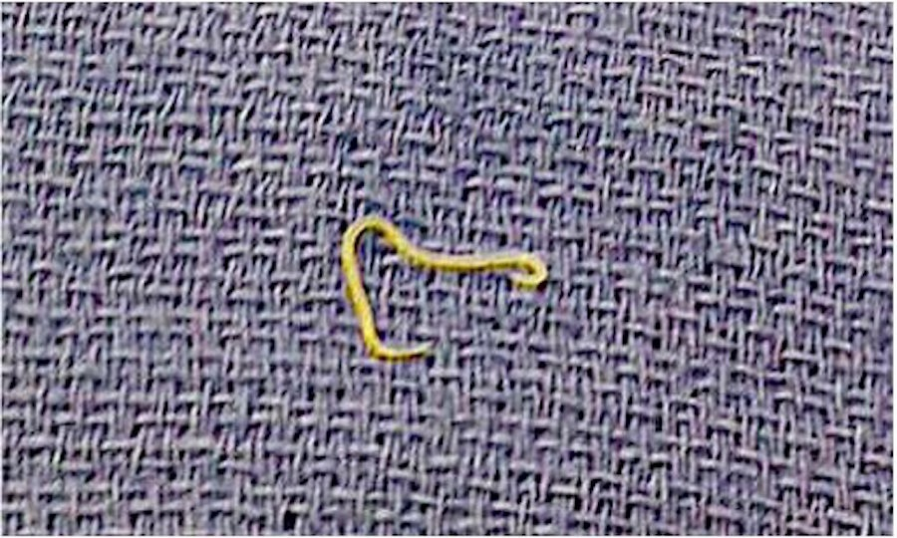
Anisakid worm from peritoneal lavage

A 37-year-old healthy female presented to the emergency room with abrupt severe epigastric pain, which progressed gradually towards the periumbilical area, associated with nausea and multiple episodes of nonbloody vomiting. On examination, she was febrile with a temperature of 100.4°F and had diffuse abdominal tenderness with guarding on palpation. The only significant laboratory abnormality included a white blood cell (WBC) count of 16,000/uL with 94% granulocytes. There were no eosinophils on differentiation. The liver profile and lipase levels were unremarkable. Qualitative beta human chorionic gonadotropin (HCG) was negative. Given paucity of objective findings, an abdominal computed tomography (CT) was done that revealed free pelvic fluid collection (Figure 1) without any other abnormality. The patient was started on intravenous meropenem empirically. She then underwent a diagnostic laparoscopy which revealed yellowish fluid in all quadrants and some induration in the lesser and greater curvature of the stomach and the ligament of Treitz. Scattered exudates were seen along the small bowel and mesentery without any perforation. No obvious source of bilious peritonitis prompted an open laparotomy, which revealed a 2 cm long and 4 mm wide, white, wriggling worm (Figure 2) in the peritoneal exudate. This was later confirmed on microscopic examination to be an anisakid nematode. The ascitic fluid had numerous WBCs but did not grow any organism on culture. On further probing, history revealed consumption of sushi at a restaurant a day prior to the presentation. The patient gradually improved with oral albendazole and was discharged without any complications.

 

## Discussion

Although the majority of cases of gastrointestinal anisakiasis are seen in Japan (2000-3000 per year), there has been a tremendous increase in the number of reported cases in the western world [3]. In the United States, about 10 cases per year were reported during the 1970s, but this could be much higher given the rising popularity of Asian cuisine. The adult anisakid worm lives in the intestine of marine mammals as primary hosts, which hatch eggs in the water consumed by various species of fish (like sardines, cuttlefish, cod fish, salmon and anchovies), that are later accidentally consumed by humans [3].

Clinically anisakiasis can present as gastric, intestinal, ectopic (extragastrointestinal) or allergic disease. Symptoms may be due to either direct penetration of the gastrointestinal mucosa or hypersensitivity to the larvae or its secretions [4]. Acute gastric anisakid infection usually presents within 12-24 hours of contaminated seafood ingestion with abdominal pain, nausea and vomiting. This is usually seen on endoscopy and removed using a biopsy clamp [5]. Intestinal anisakiasis usually exhibits as a nonspecific presentation ranging from nausea and vomiting to acute abdominal syndrome. Thus, sometimes they go undiagnosed or misdiagnosed. Another subtype, the intraperitoneal anisakiasis, is a rare presentation as described in our case, which results from larval penetration of the stomach or intestine [6]. This could sometimes also lead to pneumoperitoneum from gastrointestinal perforation. Most cases with acute abdomen due to anisakid larvae invariably undergo a laparotomy, and virtually none are diagnosed without surgery [7]. Although antibodies against Anisakis simplex using enzyme-linked immunosorbent assay (ELISA) and other immunoassay have been developed, they are often difficult to interpret and are not useful in acute emergent situations [8]. Besides direct visualization of the worm, a recent raw fish or undercooked fish or squid consumption prior to symptomology must alert physicians to suspect this zoonotic disease early and treat it promptly.

Treatment is usually hastened by removing the worm; however, some articles have shown benefit from the use of conservative therapy with albendazole in intestinal infections [9]. The best preventive strategy includes public health education to discourage consumption of raw or inadequately cooked fish. Freezing seafood to below -31°F​^ ^for 15 hours or to -4°F (or below) for seven days, per the Food and Drug Administration, is also an easy preventative measure [10].

## Conclusions

In conclusion, with the rising popularity of Japanese cuisine in the United States, we, as physicians, should consider anisakiasis as a cause of acute abdomen whenever there is a recent consumption of raw fish in the history. High index of suspicion may lead to fewer unnecessary tests and early intervention.
